# Understanding early maladaptive schemas in autistic and ADHD individuals: exploring the impact, changing the narrative, and schema therapy considerations

**DOI:** 10.3389/fpsyg.2024.1436053

**Published:** 2024-12-04

**Authors:** Liam Spicer, Emma DeCicco, Anna Clarke, Rikki Ambrosius, Ozgur Yalcin

**Affiliations:** ^1^The Cairnmillar Institute, Hawthorn East, Victoria, VIC, Australia; ^2^School of Psychological Sciences, College of Health and Medicine, University of Tasmania, Launceston, TAS, Australia; ^3^University of Western Australia, Perth, WA, Australia; ^4^The Dash - Health Hub, Perth, WA, Australia; ^5^STAND Attuned, Perth, WA, Australia; ^6^Divergent Futures, Brisbane, QLD, Australia; ^7^Deakin University, Victoria, VIC, Australia; ^8^Enable Institute, Curtin University, Perth, WA, Australia; ^9^ANIMA Health Network, Perth, WA, Australia

**Keywords:** early maladaptive schemas, EMS, autism, ADHD, attachment, unmet needs, ACES, trauma

## Abstract

Autistic/ADHD individuals are increasingly recognised as a valid minority group, with consistent research demonstrating a higher prevalence of co-occurring mental health conditions such as PTSD, anxiety, depression, substance use, and eating disorders among other mental health challenges. Due to this, there is increasing focus on the adaptations required for Autistic and ADHD individuals of current therapeutic approaches such as Schema Therapy. Particular emphasis when creating these adaptations needs to include looking at the developmental experiences, social influences, and continued adversity faced by Autistic and ADHD individuals across the lifespan, and how the narrative around Autism and ADHD within psychotherapy in general needs to change. This paper critically examines the role of attachment, unmet needs, and adverse childhood experiences in Autistic and ADHD individuals and the subsequent impact on schema development and maintenance and mental health. This will include an overview of the current literature in this area, reconsideration of understandings of Autism and ADHD, particular therapeutic considerations and adjustments and importantly discussion around the wider societal changes that need to occur to prevent schema development and reinforcement across the lifespan.

## Introduction

There is an increasing consensus within mental health disciplines on the critical need for therapies that are specifically tailored to the etiological and maintaining factors of mental health challenges. This is of particular relevance to those who are Autistic and/or have Attention Deficit Hyperactivity Disorder (ADHD), as an understanding of these factors needs to be incorporated into an adapted therapy that also accounts for their differences. Autism is a neurodevelopmental difference, defined by variations in behaviour and functioning across various domains, including social communication, repetitive behaviours and interests, and both cognitive and sensory processing (Masi et al., 2017). Similarly, ADHD, is a neurodevelopmental difference, characterised by marked variations in areas of cognitive and emotional functioning such as attention, emotional regulation, and energy levels, with studies in recent years demonstrating the vast heterogeneity of individuals who are Autistic and/or have ADHD (Masi et al., 2017; Pelphrey et al., 2011). In this paper, our emphasis on discussing both Autism and ADHD together comes from both research and clinical and lived experience regarding the overlap of both forms of individual differences, with some studies demonstrating 50–70% of Autistic individuals also being identified to be ADHD (Hours et al., 2022). In alignment with emerging research and community preferences, this paper will also use identity-first language, referring to “Autistic individuals” rather than “individuals with Autism Spectrum Disorder (ASD),” to promote a neurodiversity-affirming approach (Bury et al., 2020; Taboas, 2023).

Research consistently indicates that, compared to neurotypical individuals, Autistic and ADHD individuals experience significantly higher rates of co-occurring mental health conditions such as PTSD, anxiety, depression, substance use, and eating disorders, among other challenges (Franke et al., 2018; Lai et al., 2019). Furthermore, they are also at risk of developing what are known as early maladaptive schemas (EMS), which are deeply ingrained negative patterns of thoughts, emotions and behaviours that develop in childhood or adolescence and become perpetuated throughout their lifetime (Young et al., 2003). Several factors that may account for this increased risk of developing significant mental health issues may include underlying factors such as temperament, neurocognitive differences, attachment/unmet core emotional needs, adverse childhood experiences (ACES), trauma, and the ongoing distress from being a marginalised group, including experiences such as discrimination and a lack of understanding and support (Botha and Frost, 2020; Cherewick, 2023; Swanepoel et al., 2017). In addition, it is important to consider the potential limitations of current psychological therapeutic models, which are primarily designed for neurotypical individuals, when considering the unique needs and experiences of autistic and ADHD individuals.

The overarching aim of this paper is to examine the aforementioned factors in Autistic and ADHD individuals and the subsequent negative impact on mental health. It will include an overview of current literature in this area and specific emphasis on the need for broader societal changes that need to occur to prevent the challenges seen in this population and schema reinforcement throughout adulthood. Furthermore, we aim to provide the relevant foundational knowledge to readers and clinicians to form the basis of various therapeutic adaptations that should be considered. This paper is an important step forward in the literature, as it synthesises our knowledge of factors related to the mental health challenges in Autistic/ADHD individuals through a broader lens and provides specific guidance on how to adapt psychological treatment, more specifically Schema Therapy, for the needs of Autistic and ADHD individuals.

## Schema therapy

Schema therapy is an integrative psychotherapy approach that significantly expands on traditional cognitive-behavioural treatments and concepts (Young et al., 2003). The therapy blends elements from cognitive behavioural, attachment, Gestalt, object relations, constructivist, and psychoanalytic schools into a rich, unifying conceptual and treatment model. At the core of Schema Therapy are EMS, which represents unhealthy core beliefs about the self, others, and the world. EMS are thought to developed as a result of negative life experiences, particularly adverse childhood experiences, and unmet core emotional needs during childhood, including stability and security in relationships, safety, autonomy, opportunities for spontaneity and play, and structure and limit setting (Young et al., 2003). The assessment of EMS is conducted using the Young Schema Questionnaire (YSQ; Young et al., 2003) and was designed to capture 18 EMS that sit in five schema domains (Table 1). However, more recent developments in schema research by Yalcin and colleagues (2020, 2023) proposed a revised taxonomy of 20 EMS based on their updated YSQ instrument, the Young Schema Questionnaire-Revised (YSQ-R; Yalcin et al., 2020, see Table 1).

**Table 1:** 20 early maladaptive schemas (EMS) and associated themes as proposed by Young et al. (2003) and revised by Yalcin et al. (2020, 2021).

Schema Domains	EMS	Associated themes
Disconnection/rejection	Abandonment	The expectation that important others will eventually abandon them
	Mistrust/Abuse	The expectation that others will intentionally abuse, and manipulate them
	Emotional Deprivation	The expectation that others will not adequately meet one’s needs for nurturance and support
	Defectiveness/Shame	The belief that one is fundamentally flawed, defective, or unlovable
	Social Isolation	The feeling of not belonging or fitting into society
Impaired autonomy	Dependence	The feeling that one is completely hopeless, dependent on others and is incapable of making everyday decisions
	Vulnerability to Harm	The belief that the world is dangerous, and that disaster can strike at any moment
	Enmeshment	Excessive emotional involvement with others (usually parents) due to the belief that one cannot be happy/survive without the other results in the inability to form own identity
	Failure	The belief that one is fundamentally inadequate compared to others
Impaired limits	Entitlement	The belief that one is superior to others and is entitled to special privileges and rights
	Insufficient Self-Control	Difficulties exercising self-control to achieve goals, low frustration tolerance, and inability to control urges and impulses
Other—directedness	Subjugation	Excessive subjugation of needs to avoid punishment, abandonment, and rejection.
	Self-Sacrifice	Excessive or exaggerated expectations of duty to meet the needs of others at the expense of own fulfilment
	Approval Seeking	Excessive focus on gaining the attention, recognition, and approval of others often at the expense one’s own sense of self
Over vigilance and inhibition	Negativity	An increased focus on the negative aspects of life, whist minimising and neglecting the positive
	*Emotional Inhibition	The belief that the expression of emotion may lead to negative consequences such as harm or embarrassment to others
	Unrelenting Standards	The belief that one will be harshly criticised if they do not meet very high standards (often internalised standards) of performance or behaviour
	*Punitiveness	The belief that people, including themselves, who make mistakes should be harshly punished
EMS from YSQ-R yet to be assigned to domains	† Emotional Constriction	Excessive overcontrol of emotions due to feelings of shame and embarrassment of all emotions
	† Fear of Losing Control	A belief that dire consequences will result from failing to maintain control of emotions
	† Punitiveness (Self)	Self-directed hyper-criticalness and punishment for a person’s own mistakes, suffering, or imperfections
	† Punitiveness (Other)	The belief that others should be harshly punished and suffer consequences for indiscretions and mistakes

†EMS in the YSQ-R and *EMS not contained in the YSQ-R.

Schema therapy is known to be effective for difficult-to-treat populations when previous cognitive behavioural approaches were not yielding long-term effectiveness (Pilkington et al., 2023). Schema Therapy has demonstrated to be effective in reducing symptomatology for a wide range of clinical presentations including borderline personality disorder (Arntz et al., 2022), substance use disorders (Ball, 2007), agoraphobia (Gude and Hoffart, 2008), panic disorder (Hoffart and Sexton, 2002), depression (Carter et al., 2013) and post-traumatic stress disorder (Cockram et al., 2010; Fry et al., 2024) among others. These are all clinical presentations seen in higher rates in Autistic and ADHD individuals in comparison to the neurotypical population, demonstrating the importance of transdiagnostic interventions such as Schema Therapy (Franke et al., 2018; Lai et al., 2019).

## Schema therapy for autistic/ADHD populations

A recent scoping review by Vuijk et al. (2024) synthesised the existing research on the application of Schema Therapy to Autistic individuals, identifying only three studies that evaluate its effectiveness for co-occurring mental health conditions. The authors noted that some modifications to the Schema Therapy conceptual model, tailored for the Autistic population, have been explored, with additional literature revealing prevalent EMSs among Autistic individuals and detailing two case studies demonstrating some effectiveness for Schema Therapy with co-occurring mental health challenges in Autistic individuals. Of note to mention is the article from Bulluss (2019) due to its emphasis on integrating Schema Therapy in a more affirming way, highlighting a strengths-focused approach that values individual differences. In contrast to the research on Autism and Schema Therapy, the literature on Schema Therapy for individuals with ADHD is markedly sparse, with studies primarily documenting the EMS prevalent within this demographic (Miklósi et al., 2016; Philipsen et al., 2017). The paucity of research on the efficacy and adaptation of Schema Therapy for both Autistic and ADHD populations underscores a significant gap in developing tailored therapeutic approaches for these neuro-minority groups. There must be a holistic and multifaceted understanding of experiences from birth to adulthood in Autistic and ADHD clients when integrating Schema Therapy with this population group. Given differences in social communication style, emotion regulation, sensory processing, and executive functioning for Autistic and ADHD individuals, evidence-based treatments need to be adapted for these populations (Carbajal and Praetorius, 2020). Schema Therapy if adapted correctly, provides a promising avenue for the treatment of co-occurring mental health challenges in Autistic and ADHD individuals due to its emphasis on understanding the key patterns in which someone views themselves, and how this has developed and evolved across the lifespan. Furthermore, we argue this approach is particularly fitting due to its transdiagnostic application (Bach and Bernstein, 2019), with high incidences of various mental health challenges seen in both Autistic and ADHD individuals as highlighted. We will now explore the current literature on schemas in Autistic and ADHD individuals, before examining the relationship with various experiences across the lifespan.

## Early maladaptive schemas in autistic and ADHD populations

Research exploring EMS in Autistic individuals identified the most common EMS presenting in this group as emotional deprivation, insufficient self-control and vulnerability to harm and illness (Oshima et al., 2015). Other highly endorsed EMS included social isolation defectiveness/shame, failure, and mistrust/abuse (Oshima et al., 2015). Prevalent EMS identified in ADHD individuals include failure, defectiveness/shame, emotional deprivation, subjugation, emotional inhibition, insufficient self-control, and social isolation (Kiraz and Sertçelik, 2021; Philipsen et al., 2017). Notably, the current research is limited in the fact that no studies have explored Autism and ADHD together in various schema profiles, despite as mentioned, there being a high co-occurring overlap between Autism and ADHD. The commonly identified EMS may capture the challenges experienced by Autistic and ADHD individuals in their early and later development which can assist therapists of particular EMS to be aware of in therapeutic practice. However, it is important to consider the reality and experiences of Autistic and ADHD individuals through a wider social lens in the development and formation of schema development. As an example, the high prevalence of the social isolation schema is consistent with evidence that ADHD children and adolescents are less popular in general, have fewer positive social connections (Elkins et al., 2011) and experience more rejection than peers without ADHD (Murray-Close et al., 2010). Furthermore, insufficient self-control may reflect ADHD individuals’ differences regarding areas such as impulsivity, and completion and organisation of tasks (Silverstein et al., 2020) without person-centred approaches to learning being considered. Lastly, high vulnerability to harm EMS scores in Autistic individuals may reflect the lived realities of living with a higher incidence of medical challenges and conditions. This includes seizure disorders, hypertension, gastrointestinal illness, immune conditions, chronic sleep conditions, diabetes, POTS, and Ehlers-Danlos, among others (Croen et al., 2015; Fortuna et al., 2016). These few examples demonstrate the importance of a broader perspective in understanding schema profiles, and their influences on this population group.

The importance of understanding the factors related to schema development in Autistic and ADHD individuals is of paramount importance as it assists in guiding preventative but also treatment approaches for EMS and mental health. Research has demonstrated that increased severity of EMS in Autistic and ADHD individuals corresponds with poorer mental health (Oshima et al., 2014; Miklósi et al., 2016), with EMS posited to mediate the relationship between Autistic/ADHD traits and negative mental health outcomes (Miklósi et al., 2016; Oshima et al., 2014). As mentioned, the rates of diagnosed mental health challenges are significantly higher in Autistic and ADHD individuals, supporting the need to understand factors related to schema development. If we aim to understand the best ways of providing support to Autistic and ADHD individuals, we need to have a greater understanding of the causal and maintaining factors in schema development, and how they are reinforced and exacerbated from birth to adulthood. This includes a focus on the role of attachment, adverse life experiences and their interplay with EMS which will now be discussed.

## Attachment and unmet needs in autistic and ADHD individuals

The early social environment plays a critical role in shaping an individual’s future emotional and social development. Attachment theory, pioneered by Bowlby (1969) and expanded upon by Ainsworth et al. (1974), emphasises the enduring impact of caregiver responsiveness on attachment styles. Secure attachment, fostered by consistent and attuned caregivers, lays the foundation for emotional regulation, healthy relationships, and self-worth. Conversely, insecure attachment styles, such as anxious or avoidant, can develop in response to inconsistent or unavailable caregivers, potentially leading to greater social difficulties, emotional dysregulation (Bartholomew, 1990), and unmet core emotional needs (Young et al., 2003), which impacts on the formation of EMS.

If we start to explore the research done in this field, we can aim to not only get a greater understanding of the role of attachment in EMS development, and its association with mental health challenges, but create greater levels of awareness around factors for clinicians to be mindful of when using Schema Therapy interventions with this population. Lee et al. (2017) suggest Autistic children have differences regarding social processing and communication, potentially hindering the formation of secure attachments if parents are not understanding and receptive to meeting their specific needs in an attuned way. The development and experience of attachment in individuals that are Autistic and/or ADHD reveal the intricate and multi-faceted nature of meeting an individual’s core needs. Teague et al. (2017) illustrate this complexity, demonstrating how some parents may feel disconnected from their autistic children due to the child’s atypical engagement in eye contact, conversational patterns, and physical contact. Insensitive or dismissive caregiver responses towards repetitive behaviours or different communication styles in autistic individuals have also been revealed to create a frustrating environment for parents who do not have an understanding of being more receptive to unique needs and may contribute to insecure attachment (Green et al., 2010).

Insecure attachment styles are notably prevalent among ADHD populations, and these attachment experiences have been shown to potentially exacerbate challenges throughout childhood and into adulthood (Storebø et al., 2016; Sonuga-Barke et al., 2017). Research suggests that in children with ADHD there may be fewer positive child-caregiver interactions (Gau and Chang, 2013), particularly if the caregiver also has ADHD (Griggs and Mikami, 2011), emphasising the importance of self-understanding and support. Furthermore, inconsistent caregiver responses can lead to the development of an emotional deprivation schema and may magnify existing challenges in emotional regulation and frustration tolerance. Regarding support around emotions in childhood for Autistic and ADHD children, it has commonly been observed in clinical practice that if caregivers may not understand the emotional differences and be attuned in times of bigger emotions this may impact on the development of other highly endorsed schemas. This may include schemas such as emotional inhibition and subjugation which may be related to the common tendency to mask Autistic/ADHD traits in some individuals (Kosaka et al., 2019), as both involve suppressing or hiding one’s needs and emotions. This tendency towards putting others’ needs first and suppressing one’s own emotional responses can be continually reinforced through educational and other social environments that may not be understanding and celebrating of differences in thinking, functioning, and behaving in the world.

Additionally, the stress associated with parenting a child with a disability can impact on the mental health of parents of Autistic/ADHD children, which with a lack of sufficient support and resources exacerbates challenges with connection, ongoing support throughout childhood, and various emotional needs being met (Teague et al., 2017; Zaidman-Zait et al., 2017). Given Autism and ADHD are highly heritable, there is also a substantial risk of intergenerational trauma within these families (Craig et al., 2020; McDonnell et al., 2019), impacting parental capacity and ability to meet needs. Furthermore, rates of parental incarceration, divorce, parent mental health issues, and marital stress are higher for Autistic and ADHD children (Berg et al., 2016; Crouch et al., 2021; Schwartz et al., 2023; Walker et al., 2021; Hartley et al., 2023). This impacts parents of Autistic and ADHD children reporting they feel socially isolated, stigmatised, and unable to access or receive the support they need to best care for their child (Leitch et al., 2019; Stewart et al., 2022). A combination of all of these stressors can increase the perceived difficulty of parenting, reduce parenting quality, increase child behavioural challenges, and potentially interfere with the parent–child relationship across the lifespan (Kinnear et al., 2016; Osborne and Reed, 2010; Zaidman-Zait et al., 2017).

In exploring the impact of attachment across the lifespan, the emotional unavailability, reduced capacity, lack of support and attachment disruptions commonly experienced by Autistic and ADHD individuals are important predictors of mental health challenges and dissociation later in life (Dutra et al., 2009). These initial attachment challenges regarding various emotional needs not being met in a unique way considering the child’s differences can then be magnified throughout childhood and adolescence without appropriate understanding, support, and accommodations. For example, ADHD children are more likely to experience negatively expressed emotions and criticism (e.g., being described as ‘lazy’) from their parents/caregivers, with subsequent negative impacts on self-esteem (Dan and Raz, 2015; Musser et al., 2016; Beaton et al., 2022). In clinical practice it has been commonly observed from the authors that without an Autistic/and or ADHD individual having an appropriate understanding from others around them in their differences or reasons for certain behaviours, there can then be a tendency for these individuals to internalise messages such as “I am not trying hard enough” or “I am so lazy.” These key ongoing experiences may be large contributing factors to the higher schema scores of failures and defectiveness/shame seen in both Autistic and ADHD individuals. In looking at the impacting factors in schema development and mental health, the role of ACES for Autistic and ADHD individuals also needs to be explored.

## Exploring adverse experiences in autistic and ADHD individuals

Exploring the impact of ACEs in schema development in Autistic and ADHD clients is critical. Previous meta-analyses by May et al. (2022) and Pilkington et al. (2021) highlight associations between ACEs and EMS in adolescents and adults. For example, parental emotional abuse and/or neglect shows a moderate to high correlation with Emotional Deprivation, Mistrust/Abuse, and Social Isolation EMS in adolescents (May et al., 2022). The experiences of ACEs and the original study in this area explored the prevalence and impact of 10 potentially traumatic events occurring during childhood; physical, emotional, or sexual abuse, physical or emotional neglect, parental separation/divorce, exposure to family violence, and parental mental health, incarceration or substance use (Felitti et al., 1998). Results highlighted ACEs are correlated with a wide range of negative physical, mental, and social outcomes (Felitti et al., 1998; Walsh et al., 2019). ACEs are now widely accepted as significant risk factors for poorer life outcomes including chronic health problems, cardiovascular disease, substance use disorders, depression, unemployment, and premature death (Bellis et al., 2019; Merrick et al., 2019). A graded dose relationship has also been established whereby the higher a person’s ACE score, the more likely they are to experience worse life outcomes.

Concerningly, research consistently highlights that Autistic and ADHD individuals are at higher risk of experiencing all 10 ACEs compared to the general population (Berg et al., 2016; Crouch et al., 2021; Hartley et al., 2023; Kerns et al., 2022; Schwartz et al., 2023). In a recent meta-analysis, Autistic people were identified as more than twice as likely as non-Autistic people to experience at least one ACE (Hartley et al., 2023). Further, Autistic and/or ADHD children are more likely to have an ACE score above four compared to neurotypical children (Berg et al., 2016; Crouch et al., 2021). Physical abuse/assault is the most frequently reported ACE among Autistic children (Hall-Lande et al., 2015; Hartley et al., 2023; Rumball et al., 2021), with risk even higher for those with a co-occurring intellectual disability (McDonnell et al., 2019). ADHD children also experience physical abuse (Fuller-Thomson and Lewis, 2015; Schwartz et al., 2023) and physical neglect (McDonnell et al., 2019; Schwartz et al., 2023) at higher rates than the general population. Rates of sexual abuse are also alarmingly high (Dike et al., 2023). For example, a Swedish sample of 4,500 children, female Autistic children were found to have a threefold increased risk of sexual abuse, while ADHD children were found to have a double risk (Ohlsson Gotby et al., 2018). Due to these higher incidences of emotional abuse and/or neglect during childhood, this may lead to an increased masking of Autistic or ADHD traits as a survival response to avoid negative social appraisal (Pearson et al., 2023), further reinforcing schemas such as emotional inhibition and subjugation. Furthermore, these experiences may be significant contributing factors in the other highly endorsed schema of mistrust/abuse, as an individual’s internal template of a person and the capacity to trust them is significantly impacted by abuse experiences (Goldenson et al., 2021).

Expanded versions of the ACEs questionnaire now exist, which recognise potentially traumatic childhood events that occur in community settings, including bullying, discrimination, and neighbourhood violence (Cronholm et al., 2015). Autistic and ADHD children are also at increased risk of these types of adverse experiences in the community, particularly bullying (including physical assault, name-calling, exclusion, and other forms of emotional abuse; Hartley et al., 2023; Schwartz et al., 2023), key factors driving schemas such as mistrust/abuse and social isolation. Autistic children are more likely to experience poly-victimisation (experiencing multiple forms of maltreatment from multiple perpetrators), particularly abuse in institutional/healthcare settings (e.g., chemical/physical restraint and loss of autonomy; Kerns et al., 2022). Furthermore, Autistic and ADHD children are more likely to live with families experiencing economic hardship, social disadvantage (Berg et al., 2016; Crouch et al., 2021; Walker et al., 2021), and experience prejudice and discrimination (Pearson et al., 2023) due to their differences demonstrating a complex interplay of both individual and societal level adversity. As highlighted, clinicians and other individuals supporting Autistic and ADHD individuals need to consider the ongoing dynamic interplay between individual, and societal experiences, and how both attachment and adverse experiences need to be viewed from this wider societal lens. The next section of this paper will discuss this more explicitly, providing further understanding of Autism and ADHD in line with current paradigms, with treatment considerations with all of this in mind to follow.

## What needs to change and adapted schema therapy approaches

Historically, bio-medical models have focused on the core features of Autism/ADHD, treating them as the pathological cause of symptoms and distress (Linden et al., 2023; Pellicano et al., 2014). However, behavioural approaches that aim to alter individuals to fit neurotypical standards can, in some cases, be harmful (McGill and Robinson, 2020), and largely ineffective in improving mental health and quality of life outcomes (Linden et al., 2023; van Heijst and Geurts, 2015). We argue, in line with increasing research, discussion upon experts in the field, and importantly learning from and valuing the importance of lived experience accounts, that Autism and ADHD should be viewed and considered in a way that is in line with the Neurodiversity Affirming Paradigm (Walker, 2014). The Neurodiversity Affirming Paradigm is an expansion and evolution of the disability rights movement, centring on a more affirming view of cognitive diversity, and valuing the importance of individual differences (Pantazakos and Vanaken, 2023). This social shift, and significant step forward in collective social attitudes and perceptions in simple words states that individual differences should be viewed as valuable forms of human diversity, rather than being pathologised or medicalized which has been the predominant view historically (Walker, 2014). One such example is the use of language when referring to individuals who are Autistic and/or ADHD. For example, in one study Taboas (2023) demonstrated that overwhelmingly Autistic adults preferred identity first language such as “Autistic person,” in comparison to person-first language such as “person with Autism” or “person with ASD.” Building from this example, referring to Autism as a Disorder, or labelling someone as low or high-functioning is not considered to be in line with the Neurodiversity affirming approach. Instead, more appropriate choices of language in replacement can be “Autistic Person,” and “high or low support needs.”

**Figure 1:**
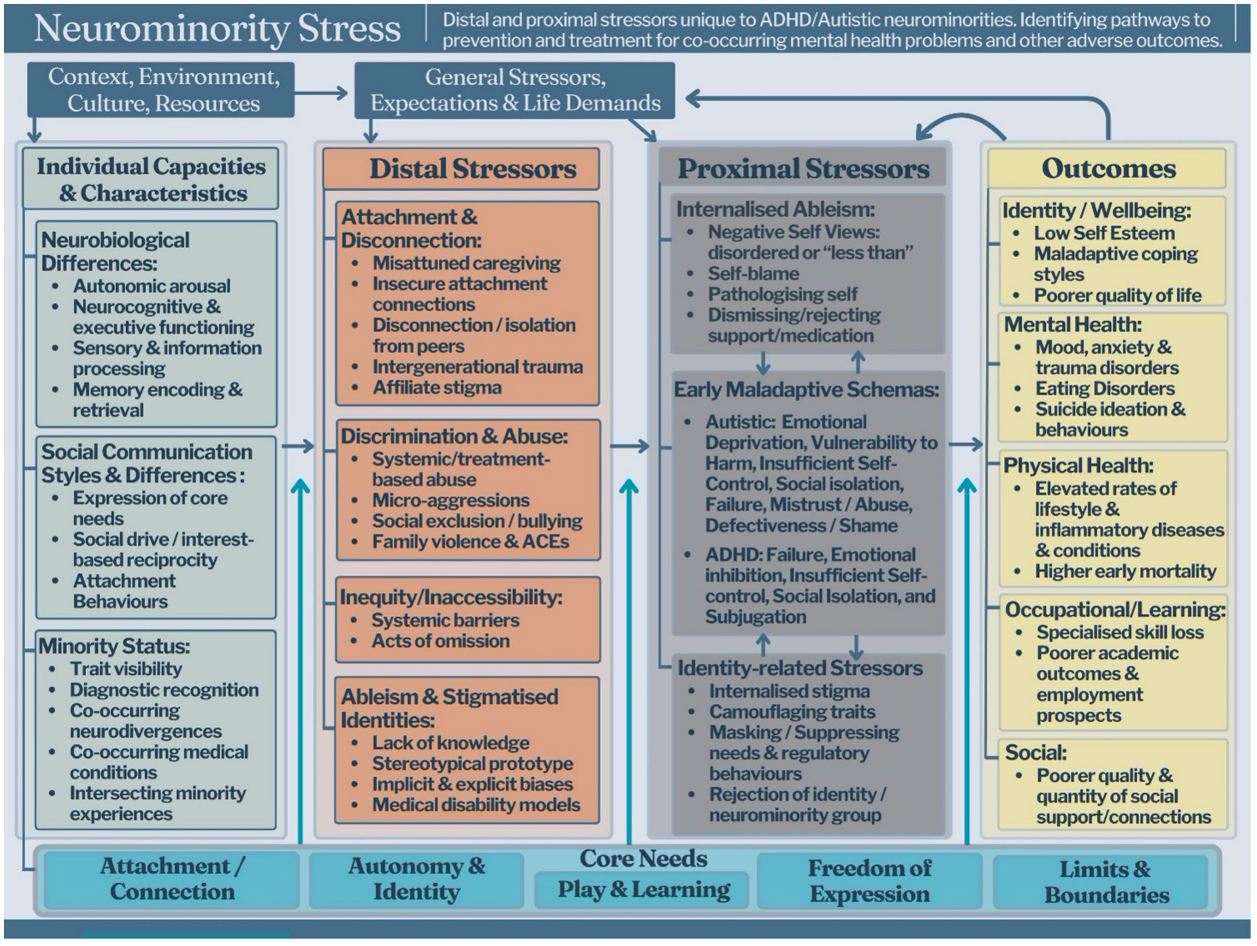
Neurominority stress model (Copyright—STAND Attuned, 2024, reproduced with permission).

Reconceptualising Autistic and/or ADHD individuals as a neurominority, with core features that reflect ontogenetic adaptations to person-environment fit (Cherewick, 2023; Johnson et al., 2015), has important implications for understanding the prevalence of and pathways to co-occurring mental health issues (Botha and Frost, 2020). This neurominority exhibits heterogeneous differences and strengths across neurocognitive functioning, learning, autonomic arousal, sensory processing and social-communication styles (Milton, 2012; Djerassi et al., 2021; Bellato et al., 2022; Piccardi and Gliga, 2022; Patil and Kaple, 2023; Schwartz et al., 2023; Scheerer et al., 2024). Autistic/ADHD differences are shaped by the bi-directional interplay between genetically driven neurology (e.g., temperament or stress responses) and environmental factors (e.g., support; Cherewick, 2023; Johnson, 2017; Swanepoel et al., 2017). Therefore, the developmental trajectories of Autistic and/or ADHD individuals, including their psychological well-being, largely reflect biopsychosocial processes related to the person-environment fit across their lifespans (Botha and Frost, 2020; Cherewick, 2023; Swanepoel et al., 2017). This is demonstrated by Figure 1, which is an adapted version of Meyer’s Minority Stress Model. This adapted version demonstrates the unique stressor processes faced by neurominorities (Botha and Frost, 2020; Meyer, 2003) increasing their vulnerability, and exacerbating various emotional and mental health challenges.

This viewpoint acknowledges the multifaceted and complex range of both individual factors, life experiences and socioenvironmental factors that impact on the mental health of Autistic and/or ADHD individuals. Importantly, these frameworks and greater understanding of both individual and societal influences on neurominorities can ensure therapy approaches are adapted to meet the needs of the specific populations we are working with. Born with neurological “starting state” differences (Johnson, 2017), neurominorities navigate social and physical environments predominantly tailored for neurotypical individuals. Consequently, Autistic/ADHD individuals often face significant person-environment mismatches, encountering distal stressors through unrecognised, pathologised, or unsupported differences (Hayes et al., 2018), leading to sustained challenges in core psychological needs being met. Further distal stressors are experienced through various forms of indirect discrimination (e.g., inequitable learning environments, lack of parental support), stereotypes, as well as higher rates of direct victimisation, stigmatisation and other forms of abuse (Aguado-Gracia et al., 2021; Pfeffer, 2016; Schwartz et al., 2023). The chronicity, intensity, and valency of distal stressors are critical in shaping an individual’s self-perception and worldview. This often manifests as internalised ableism (Morgan, 2023), as well as anticipated discrimination and identity-related stressors such as shame and identity concealment (Kiraz and Sertçelik, 2021; Oshima et al., 2024; Someki et al., 2018; Turnock et al., 2022). These proximal stressors are strikingly broad, negative emotional and cognitive patterns that are associated with poorer mental health outcomes and directly align with the development and perpetuation of EMS (Kiraz and Sertçelik, 2021; Oshima et al., 2021; Philipsen et al., 2017).

EMSs and subsequent EMS-reinforcing coping responses can be conceptualised as proximal stressors that predispose the individual to mental health issues (Kiraz and Sertçelik, 2021; Philipsen et al., 2017; Someki et al., 2018). Such perspectives account for the mounting evidence that mental illness and psychological distress are neither inevitable consequences nor directly tied to the core features of ADHD and Autism (Botha and Frost, 2020; Linden et al., 2023). Indeed, interventions that seek to reduce core autistic features are not consistently predictive of mental health outcomes (Linden et al., 2023) and may result in the loss of strengths including specialised skills and passions (Eigsti and Fein, 2013; McGill and Robinson, 2020), and reinforce masking and schema development (e.g., “There’s something wrong with me”). Similarly, interventions that target core ADHD features are significantly less successful than treatments that target co-occurring emotional challenges (e.g., Payne et al., 2022). The poor efficacy of current interventions may be in part explained by an erroneous characterisation of core Autistic/ADHD features as proximal stressors. Furthermore, neuro minorities face significant treatment barriers from clinicians with inadequate knowledge and experience, as well as from failures to appropriately adapt therapies to Autistic and/or ADHD needs (Adams and Young, 2020). Therefore, Autistic and/or ADHD individuals with co-occurring mental health problems face further exposure to distal stressors in the form of mis tuned neuro-normative-based therapeutic interventions that fail to understand and meet their core needs.

**Figure 2:**
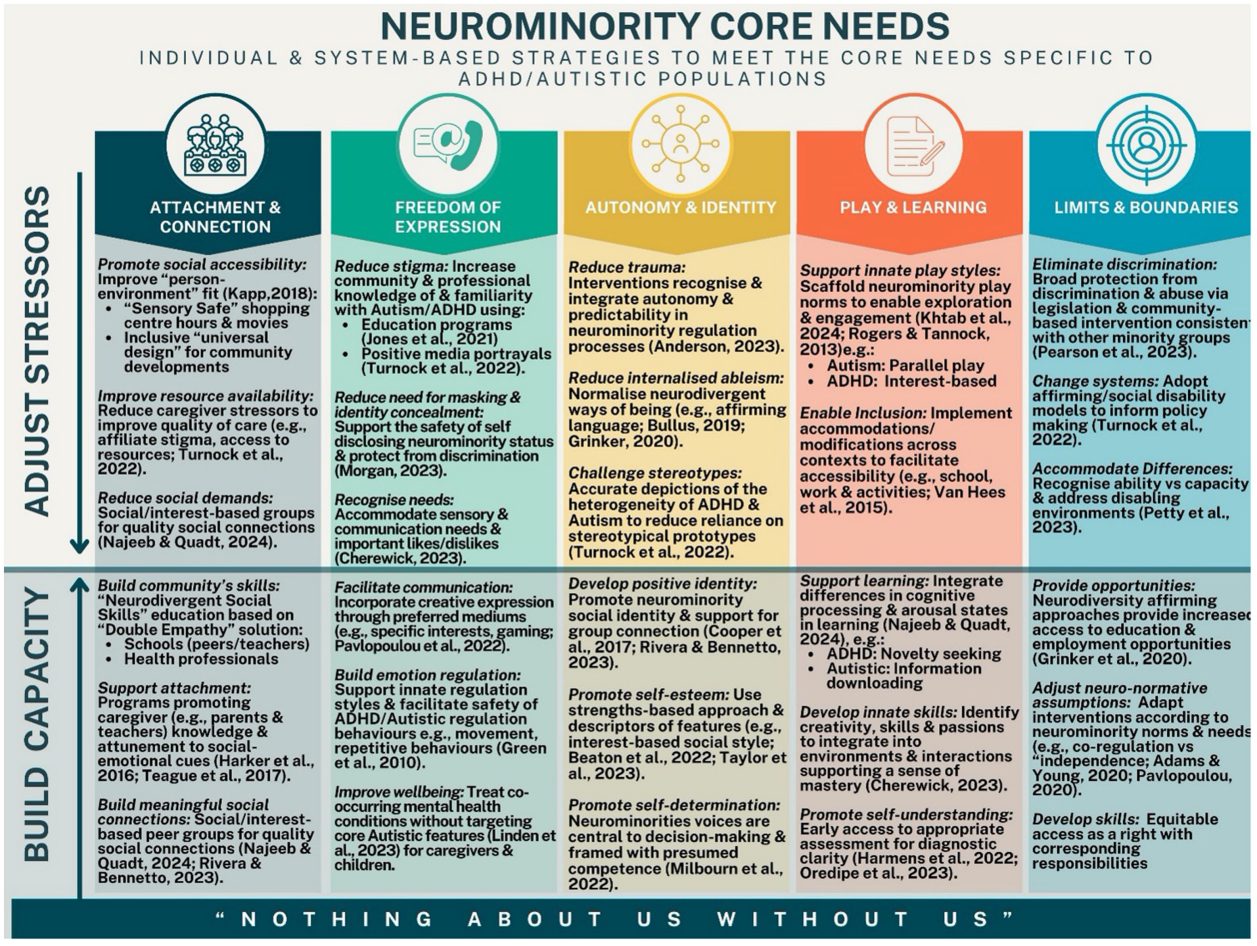
Processes proposed to better meet neuro minority core needs.

Identifying processes that positively influence the developmental trajectories of neurominorities is a critical step for the proactive cultivation of mental well-being across the lifespan (Linden et al., 2023; Del Rosario et al., 2023; Najeeb and Quadt, 2024). Although research on this topic is in its infancy, the available evidence of potentially beneficial processes can be directly mapped against core psychological needs. Figure 2 summarises the growing body of evidence demonstrating improved developmental and psychological outcomes where interventions effectively target the underlying processes that facilitate the meeting of Autistic and/or ADHD-specific core psychological needs.

Prominent examples including improving caregiver/child attunement (attachments; Beaudoin et al., 2019; Del Rosario et al., 2023; Whitehouse et al., 2021; Whitehouse et al., 2020), promoting innate strengths and differences, (Cherewick, 2023; Taylor et al., 2023), strengthening neurominority identity (autonomy; Rivera and Bennetto, 2023), as well as passions and interests (play and fun; Cherewick, 2023; Milbourn et al., 2022). Specifically, we hypothesise that satisfying core emotional needs through both individual supports, but wider socioenvironmental change can significantly mitigate the formation of EMSs and related mental health challenges (Grażka and Strzelecki, 2023) across the lifespan. The development of effective preventative and intervention approaches relies on distinguishing and addressing the pathways to co-occurring mental health problems in Autistic and ADHD populations (Bergman et al., 2020; Botha and Frost, 2020). These will now be discussed in subsequent sections with reference to the Schema Therapy model and its core emotional needs emphasising the importance of these being met through individual experiences, but also through wider social acceptance and change. Furthermore, specific sections on parenting and therapeutic adaptations will also be covered.

## Meeting core needs of autistic and ADHD individuals at an individual and societal level

If we want to optimise our capacity for creating change for Autistic and/or ADHD individuals, we need to expand our framework and approach in understanding and meeting core needs across all areas of life. We are now going to discuss and highlight the five core needs of the Schema Therapy Model, with reference to how these needs can be met through not only individual support but through wider socioenvironmental change.

### Core need: attachment and connection

Systemic change requires a deep understanding of the drivers of distal stressors, a process crucial for developing effective strategies that address harmful cultural norms, legislative gaps, institutional practices, economic barriers, and misinformation (Botha and Frost, 2020; Pearson et al., 2023). Transforming stereotypes and discrimination involves shifting cultural narratives, including the information people are actively taught (e.g., education programmes, conferences) and passively exposed to (e.g., media portrayals; Jones et al., 2021; Turnock et al., 2022). Employing accurate representations of the heterogeneity of neurominority features in entertainment media serves to increase awareness of diversity and reduce reliance on outdated stereotypical prototypes (Turnock et al., 2022). Such initiatives promote awareness of nuanced neuro minority differences, which may improve early detection, diagnosis and vital access to support, factors associated with improved outcomes (Harmens et al., 2022; Oredipe et al., 2023). Furthermore, understanding neuro minority social-communication and behavioural differences aids the attunement, support and caregiving processes implicit in interactions with both formal (e.g., psychologist) and informal (e.g., parent) care providers, ultimately improving the quality of connections and support (Harker et al., 2016; Teague et al., 2017).

### Core needs: freedom of expression of valid needs and wants

Effecting meaningful change requires that the perspectives of minority groups are not only heard but are prioritised. Inclusive stakeholder representation and participatory decision-making are fundamental in addressing systemic issues for neurominority groups (Milbourn et al., 2022). This approach is exemplified in the neuro minority context by the mantra “nothing about us without us,” widely adopted among advocacy groups to emphasise the importance of empowerment and self-determination (Najeeb and Quadt, 2024; Turnock et al., 2022). Elevating Autistic and ADHD voices is critical in combating the misinformation that perpetuates inaccurate stigmas and ableist rhetoric and contributes to the development of positive group identities.

### Core needs: autonomy and identity

Embracing a shared social identity within supportive groups is associated with higher levels of self-esteem and reduced internalised stigma (Najeeb and Quadt, 2024; Taylor et al., 2023). This sense of belonging and community is crucial for fostering a positive social identity among neurominorities, ultimately contributing to the quality of their social connections, mental health and overall well-being (Najeeb and Quadt, 2024; Rivera and Bennetto, 2023). As healthy self-identity is strongly associated with higher levels of wellbeing, providing support for people to authentically express themselves, celebrating unique strengths and qualities, and creating societal change regarding perceptions towards neurodiversity is critical (Kapp, 2018; Najeeb and Quadt, 2024; Oredipe et al., 2023; Pavlopoulou, 2020; Tesfaye et al., 2023). Regarding providing support for the development of autonomy, individual and societal attitudes need to shift, particularly internalised ableism and how it may take away opportunities for independence and skill development, potential barriers in healthy self-identity.

### Core need: play and learning

Of particular importance in improving neurominority outcomes, is the critical need to enhance the quality and availability of caregiving through increased support and resources. Interventions that improve mental and physical health literacy and well-being are vital for fostering connection and safety within and between families. Recent studies highlight how positive childhood experiences can mitigate the effects of adverse childhood experiences (ACEs), with activities like family discussions, support during challenges, community participation, and feelings of safety showing beneficial effects (Bethell et al., 2019; Crandall et al., 2023; Morris and Hays-Grudo, 2023). Therefore, providing neurodiversity-affirming education, parental support, and addressing public stigma are essential for enabling Autistic and ADHD families to access these positive and protective experiences, which help prevent and reduce the development of early maladaptive schemas across the lifespan.

Challenging societal norms that elevate neurotypical features and behaviours as the ‘expected standard’ is crucial for normalising neurominority ways of being (Botha and Frost, 2020) and reducing stigma-related stressors (internalised, affiliate and anticipated). Fundamentally, affirming neurodiversity means the ‘goalposts’ are no longer neurotypical; instead, the game centres on inclusivity, diversity, and individual strengths. This facilitates the abandonment of coercive conformity practices (Anderson, 2023; Pavlopoulou et al., 2022) and standardises the incorporation of neurominority needs into environment designs (e.g., universal design; Kapp, 2018; Narenthiran et al., 2022), which is critical in improving access to education, employment, and social engagement opportunities (Grinker, 2020). Furthermore, adopting affirming, strengths-based approaches to neuro minority features is associated with better quality of life, subjective well-being, and lower levels of anxiety, depression, and stress (Taylor et al., 2023).

### Core need: reasonable limits

Improved mental health and quality of life outcomes in neuro minorities requires health professionals to revisit bio-psycho-social frameworks that consider the broader ‘person-environment fit’ moving beyond the medical disability model that targets only the individual (Najeeb and Quadt, 2024). Broad systemic changes are essential to limit neuro minorities’ exposure to distal stressors in the form of discrimination, abuse, inequity, lack of accessibility, ableism, and stigmatised identities. Merely providing therapy to the individual without addressing these broader issues is akin to ‘patching up patients and sending them back to the battlefield’. While such interventions may offer temporary stabilisation, they are insufficient for effecting lasting change if the harmful environments contributing to the individual’s distress remain unaddressed. This approach is in line with anti-oppressive therapy practices, where therapists take a more active stand in the promotion, encouragement, and removal of barriers in systematic social change.

Recognising and meeting the core psychological needs of neurominority individuals is paramount in preventing the development of EMSs during childhood (Young et al., 2003), and also serves as a crucial foundation for designing effective mental health interventions tailored to Autistic/ADHD populations. These interventions must be complemented by initiatives that effectively address neuro-minority-specific distal stressors (e.g., accommodations and modifications to support equitable access to education), as well as proximal stressors (e.g., internalised ableism). Schema therapy, grounded in the understanding of early developmental experiences and the significance of meeting core needs, is placed to be an overarching and adaptable framework to utilise with Autistic and ADHD individuals. We are now going to discuss the application of this Schema Therapy framework firstly in the context of parenting more specifically as a key preventative measure, and secondly how we can utilise an adapted Schema Therapy framework in therapy settings.

## Prevention of schema development through parenting

As highlighted in our discussion of the meeting of core needs for Autistic and ADHD individuals, parental/child attunement can be a key focus point for reducing the development of EMS. Teague et al. (2017) suggest that both parental sensitivity and insight into their child’s needs promote secure attachment in individuals who are Autistic, and in addition to the strategies mentioned which can be promoting of positive needs meeting, the utilisation of a Schema Therapy framework may further enhance the capacity for change in this important area. One such approach is the Good Enough Parenting approach proposed by Louis et al. (2021). This innovative, Schema Therapy focused approach provides a more rich and nuanced understanding of key factors which may impact on parental attunement and positive parenting patterns. The parenting programme involves a dual-focus approach on the prevention of EMS, but also importantly the enhancement of early adaptive schemas via parent training (Louis et al., 2021). Early adaptive schemas are proposed to arrive from positive parenting patterns, where emotional needs are met through an attuned and connected relationship between caregiver and child (Lockwood and Perris, 2012). Similar to EMSs, certain categories in which these schemas fall under have been proposed including Connection and Acceptance, Healthy Autonomy and Performance, Reasonable Limits, and Realistic Standards and Reciprocity. The four clusters of early maladaptive and adaptive schemas appear to run in parallel, supporting the theoretical basis of both schema therapy and the Good Enough Parenting early intervention.

The Good Enough Parenting programme uses a broader and more nuanced range of parenting constructs that are grounded in schema theory; a theory that has clinical origins, and therefore a better grasp of maladaptive and adaptive patterns. There are seven adaptive parenting patterns known including: Autonomy Granting, Autonomy Support, Confidence and Competence, Dependability, Emotional Nurturance and Unconditional Love, Intrinsic Worth, Playfulness and Emotional Openness. The parenting programme also utilises 10 maladaptive parenting patterns known as exasperation interactions and labelled as: Competitiveness and Status Seeking, Over-Control, Disconnection and Rejection, Neglect and Insufficient Guidance, Emotional Inhibition and Deprivation, Intrusiveness and Exploitation, Overprotection and Overindulgence, Punitiveness and Abuse, Social Exclusion, Undependability, and Irresponsibility (Louis et al., 2021). This Schema Therapy framework for parenting acts as the foundation for the subsequent parent support and training, which can be adjusted as necessary for parenting Autistic and/or ADHD children. Of particular relevance to note is the programme includes parents becoming more aware of their own EMS patterns, a significant strength as this awareness can allow greater parental regulation, attunement, and healthy management of challenges that may come up in the parenting space. As an example, if a parent themselves has a failure schema and their child who may not be getting the appropriate support at school is having challenges, there may be a greater tendency for the parent to internalise these challenges as a response to their own parenting, and they may in response be critical towards their child resulting in the child possibly developing this schema themselves. Furthermore, as another example from clinical practice, if a parent has an abandonment schema, and also may hold internalised ableist beliefs towards Autism and ADHD this may result in an enmeshed relationship where the child’s autonomy and independence has not been encouraged and promoted. Although further discussion of this is warranted and will be the subject of future discussion, it is beyond the scope of this paper to specifically describe all components of the programme and the subsequent adjustments for this population group. However, it is worthwhile mentioning to further illustrate our message that preventative approaches at the family and societal level is critical moving forward to prevent the ongoing patterns of significant mental health challenges for Autistic and/or ADHD individuals across the lifespan.

## A need for a new way in therapy

Adapting our therapeutic approach when working with Autistic and/or ADHD clients is a vital next step for all clinicians to ensure better meeting of needs in a therapeutic context, and a general therapeutic atmosphere of acceptance and inclusion is created. The meeting of core psychological needs, complemented by interventions directed at reducing distal and proximal stressors, presents a promising avenue for improving mental health and quality of life outcomes in Autistic/ADHD neurominorities. One such approach is the recently launched Schema Therapy Affirming NeuroDiversity Model (STAND Attuned) (DeCicco et al., 2023) which specifically supports therapists in attuning to, comprehending, and addressing the treatment needs of Autistic/ADHD individuals seeking treatment for co-occurring conditions or distress, without targeting core ADHD/Autism features. Two of the authors of this paper (E.D, R.A) are involved in the development of the STAND Attuned model with further developments and evolvement of this approach currently underway. In the next section of this paper, various therapeutic adaptations will be discussed, which are not specifically related to the STAND Attuned model but draw on the authors collective experience of over 50 years working with Autistic/ADHD individuals in varying capacities. This section below also draws on the lived experience of the authors in being Autistic and/or ADHD themselves and being involved in this area in other varying capacities such as teaching, training, and research. It also is informed by continually building research supporting the components and principles of the Neurodiversity Affirming Paradigm, and National Guidelines such as the Autism CRC in Australia (Trembath et al., 2023). We aim from the previous sections discussed in this paper that some of the primary knowledge and an update in the framework on how Autism and ADHD should be viewed has provided a solid platform for the basis of the proposed therapeutic adaptions we will now discuss. Although this area could be covered in greater detail, we argue that the following section covers some of the key considerations and components to applying Schema Therapy in a more Neurodiversity Affirming way.

## Therapeutic adaptations

In our Schema Therapy approach, consideration can be given to all of the core components and processes of therapy and how they may need to be adapted to meet the unique differences and needs of our clients that are or may be Autistic and/or ADHD. The following section is going to include a discussion of some of the considerations and possible adaptations that may be needed with Autistic and/or ADHD clients across three core components of Schema Therapy: the assessment phase, the therapy relationship including limited reparenting, and the various schema change techniques such as imagery rescripting or chairwork. It is important to note that no particular adaption should just be applied because someone is Autistic and/or ADHD, but it can be used for ideas and areas of exploration to best suit the needs and differences of the clients we are supporting. Furthermore, this is not an exhaustive coverage of all the suggestions, but a range of some general principles and clinical considerations for integration into clinical practice.

### Assessment phase

The assessment phase of therapy forms the foundation to create a shared conceptualisation with a client, understand their needs, treatment goals, and to start to build a therapeutic connection, which is an ongoing and evolving process within the course of therapy. Regarding some considerations for assessment with Autistic and/or ADHD individuals, significant emphasis needs to be placed on being creative, adaptive, and flexible with our assessment process, involving both obtaining qualitative and quantitative information. As an example, many of the commonly utilised questionnaires for Schema Therapy such as the Schema Mode Inventory (SMI) may have questions worded in ways that may not suit the communication or cognitive differences of an Autistic and/or ADHD individual. Furthermore, Likert scales, and having to choose an item when there is no reference to a specific time frame may be challenging for some individuals, therefore completing assessments together, or providing opportunities for clarification can be important. Lastly, our assessment needs to be one which encompasses the whole range of factors, and areas of life that may impact on Autistic and/or ADHD individuals. This includes attachment, trauma, experiences of adversity, social and interpersonal trauma, intersectionality factors, strengths, special interests, Autistic/ADHD identity, connection to the Autistic/ADHD community, the current environments in their life, and the degree to which they may experience disability in these environments. This also involves the possible need of explaining in greater detail why we are asking such questions and doing various assessments, linking this to their current clinical goals and presentation of mental health challenges.

In our assessment phase, this can be an opportunity to work together with the client to find ways of what methods and ways of learning may suit them best. For example, are they a visual person, and if so, can you draw out your schema and mode map together in a more creative way. These simple adjustments can allow building of connection through meeting needs specific to that person and can provide valuable information around how the implementation of change techniques may be best employed in future sessions. In line with a strengths-focused and neurodiversity-affirming approach, we can also aim to understand positive schemas as a part of the overall therapeutic picture. Research by Videler et al. (2020) demonstrated that focusing on positive schemas can be an avenue to create a positive therapeutic environment, a greater therapeutic relationship, can orient to the person to positive aspects of life, and also can help introduce experiential techniques later on in therapy. Another common area of consideration in this space, is being aware of the language that we are using and finding language that works for the client. For example, clients might prefer to just say “part” of me when referring to schema “modes” or have a specific label for their schema or mode in their own language. Furthermore, in our explanation of the Schema Model, ensure we are adapting this explanation to ensure it is being understood in a way that makes sense for this client. In this phase is also when we can encourage and promote the discussion of special interests as this process can enhance a sense of connection, allow an individual to feel more comfortable, and can be modelling an acceptance of differences with our identity. These special interest areas can then be integrated into future therapeutic work if relevant, including in Imagery exercises, doing chairwork, or having a reference point for healthy modes encompassing important values for the client based on what is meaningful to them and how they want to live their life.

### The therapeutic relationship

Of significant importance to the Schema Therapy model, is the therapy relationship and in particular the implementation of limited reparenting. The therapeutic relationship has been consistently discussed, and demonstrated to be a core tenet of psychotherapy, and explain variance in treatment outcomes (Norcross and Lambert, 2018). For example, meta-analyses have revealed that 20–30% of variance and outcome in therapy can be attributed to therapist factors, and with complex clients this being suggested to be much higher around the 50% mark (Cloitre et al., 2004; Norcross and Lambert, 2011; Cronin et al., 2014). There are many definitions of the therapeutic relationship, however general consensus agrees and is often characterised by a connection characterised by emotional warmth, friendliness, safety, trust, understanding, and a shared conceptualisation of history and goals (Bordin, 1979). In the Schema Therapy Model, heavy emphasis is placed on the role of the therapeutic relationship, and the role of limited reparenting which can be defined as the establishment of a secure attachment with the therapist, within the boundaries of a professional relationship.

If we start to look at the application of the therapy relationship with Autistic and ADHD clients, there are two key therapist factors that need to be of key consideration. The first one is knowledge of Autism and ADHD based on recent understandings of these individual differences as discussed. Secondly, therapists need to go through a process of self-reflection to be aware of their own explicit and implicit biases, and prejudice which may directly, or indirectly show up in the therapeutic space. This is of particular importance, as research has demonstrated that Autistic individuals report feeling understood as a key component of effective therapy, yet often face experiences of feeling misunderstood in health and medical settings (Radev et al., 2024). If we do not view Autism through an affirming lens, communicate this to clients with our words and our approach, and do not manage our own biases, how do we expect to develop a solid therapeutic relationship that is going to be meeting all of their needs. These challenges can be further magnified if our work with Autistic/ADHD clients may not be as effective as with other clients or other obstacles come up, as our own schema activation may occur. This has been demonstrated in previous research by Pilkington et al. (2022) to have various negative impacts on our therapeutic work, and therefore is an important element to be aware of through an ongoing process of mindful self-reflection.

The role of self-disclosure in schema therapy is often an applied component of limited reparenting to create a sense of connection, normalisation, and being there together as two humans. Neurodiversity affirming modes of communication involves self-disclosure (Danzer, 2018), and for Autistic/ADHD clients that may have their own internalised ableism, biases, and judgements about Autism and/or ADHD self-disclosure can assist in creating community in the room (Rainsford, 2023) whereby differences are normalised, acknowledged, and celebrated. This can also help with the facilitation around discussions of areas related to the experience of being Autistic and/or ADHD such as sensory differences, executive functioning differences, special interest areas, stimming behaviours, and other components of being an Autistic and/or ADHD person. These behaviours can all assist in building up a positive Autistic/ADHD identity, which has been shown to be protective against mental health challenges. Lastly, regardless of if you are Autistic and/or ADHD yourself as a therapist, promoting behaviours which align with your clients’ individual differences can help in establishing trust, safety, and a sense of authenticity within the therapeutic space. For example, encouraging and promoting the discussion of special interest areas, understanding and factoring in their sensory differences into the therapeutic room, and delivery of therapy, and encouraging stimming behaviours as a valid form of sensory and emotional regulation (Camin et al., 2024).

### Change phase

In consideration of some adjustments in the Change phase of Schema Therapy, consideration needs to occur for the differences in emotional, cognitive, sensory, and communication differences. As an example, the therapeutic technique of chairwork may seem too abstract for some individuals, or they may have differences regarding imaginal capacity so being able to imagine a “part” of them or someone else in the chair to say what they need to express could be challenging. Due to these possible differences, certain adaptations could include using physical items which represent the “part” of them or person they need to express their feelings to, engaging in chairwork in a more imaginal sense within their mind, or doing video recordings to then be able to show to people in their life if needed, or for general therapeutic benefits. Similar adaptations can be applied with Imagery work, where we may need to make something more visually initially to get the client to connect emotionally with the memory or experience or may need to adjust our imagery to be more somatic overall in the absence of strong visual imagery. These considerations can ensure we are delivering the intervention in collaboration with the individual’s differences in aim to make it the most positive and impactful experience. We may also need to spend specific time in the change phase working on the impact of oppressive social forces on Autistic and/or ADHD individuals. For example, as similar with other minority groups such as the LGBTIQA+ population, we can utilise chairwork with modes such as the sociocultural critic (Cardoso et al., 2023) or as proposed by the STAND Attuned Group the Ableist Critic (DeCicco et al., 2023). By using Schema Therapy experiential exercises to work on internalised biases and ableist views individuals may hold themselves in these creative ways this can aim to remove a barrier in positive identity development and overall health and wellbeing. Although further discussion is warranted and will be the subject of further work in this area, this is a brief introduction to some adjustments and considerations that may be useful when working with Autistic and ADHD individuals in your Schema Therapy practice.

## Conclusion

This paper highlights the critical interplay between the innate characteristics of ADHD and Autistic populations and the unique array of distal stressors they face, which we hypothesise to contribute to unmet core psychological needs, the development of maladaptive schemas and co-occurring mental health difficulties. Reconceptualising core neurominority features as affirmed aspects of neurodiversity, rather than proximal stressors, is essential for informing preventative measures and interventions aimed at improving mental health outcomes. It is evident that processes facilitating the fulfilment of neurominority core needs are necessary, which involves transforming stereotypes, challenging norms, normalising diversity, improving accessibility, and building unique strengths and capacity. These areas fundamentally can be preventaive actions in schema development, and ultimately improve health and wellbeing, and quality of life. Furthermore, schema therapy-focused parenting support, and therapeutic intervention based on this framework is a significant step forward in this important area. This transformative approach emphasises the importance of adapting mental health interventions to meet the unique needs of neurominorities including the critical role of societal structures in either exacerbating or alleviating mental health challenges. This paper covered some of these key considerations, encouraging adaptability, creativity, and flexibility within the therapy space. Further research on Schema Therapy with Autistic and ADHD individuals is needed. We hope from our discussions not only does it assist clinicians with learning and incorporating these adjustments into their therapeutic practice, but also, we hope that future research in this space is conducted in line with Neurodiversity affirming principles. Fundamentally, this approach involves changing the narrative to recognise and value neuro-minorities as integral threads woven into the societal fabric. By embracing these changes, we can build greater capacity within these communities and enhance overall outcomes to improve the quality of life for Autistic and/or ADHD individuals.

### Positionality statement

The authors are all Autistic and/or ADHD clinicians, researchers, and academics. Incorporating voices with lived experience is essential for producing accurate, ethical, and impactful mental health research that genuinely reflects and addresses the needs and realities of these communities.
